# Managing Older Adults With Cognitive Impairment: An Interprofessional, Standardized Patient Approach

**DOI:** 10.1093/geroni/igaa057.033

**Published:** 2020-12-16

**Authors:** Shaun Varrecchia, Carol Maritz, Colleen Maher, Megan Strauss

**Affiliations:** University of the Sciences, Philadelphia, Pennsylvania, United States

## Abstract

Several professional organizations have called for increased preparation of health professionals capable of working with older adults, including those with cognitive impairment. Standardized patients (SP) are often used in interprofessional education (IPE) in the health professions, but limited data exists to support their use when teaching about the care and management of older adults with cognitive impairment. The purposes of this project were to: 1) develop, implement, and assess an interprofessional standardized patient exercise involving physical and occupational therapy students and 2) to evaluate students’ perceptions of a SP encounter on relevance and utility to patients with cognitive impairment. 88 students representing physical therapy (DPT) and occupational therapy (DrOT) were assigned to interprofessional teams to evaluate an SP portraying an older adult with cognitive impairment. At the conclusion of the session the SP provided the group formative feedback. Student teams then completed an assignment to develop a collaborative intervention plan and addressed questions about roles and responsibilities and communication/teamwork. Pre-/post- surveys focusing on the knowledge of roles and responsibilities, communication, and teamwork were completed by all students. Students also completed an evaluation about the SP experience. Results demonstrated student agreement to understanding the role of the other’s profession improved 28.67%; being comfortable communicating with the geriatric population improved 27.31%; and working in interprofessional teams can improve geriatric patient care improved 32.11%. These findings demonstrate that use of SPs has several advantages in teaching students how to work and communicate with individuals with cognitive impairments as an interprofessional team.

